# Structural Basis for Design of New Purine-Based Inhibitors Targeting the Hydrophobic Binding Pocket of Hsp90

**DOI:** 10.3390/ijms21249377

**Published:** 2020-12-09

**Authors:** Sang Chul Shin, Ashraf K. El-Damasy, Ju Hyeon Lee, Seon Hee Seo, Ji Hyun Kim, Young Ho Seo, Yuri Lee, Ji Hoon Yu, Eun Kyoung Bang, Eunice EunKyeong Kim, Gyochang Keum

**Affiliations:** 1 Biomedical Research Institute, Korea Institute of Science and Technology (KIST), Hwarangro 14-gil 5, Seongbuk-gu, Seoul 02792, Korea; scshin84@kist.re.kr; 2 Center for Neuro-Medicine, Brain Science Institute, KIST, Seoul 02792, Korea; ph_karem2000@mans.edu.eg (A.K.E.-D.); juhyeonlee85@gmail.com (J.H.L.); shseo@kist.re.kr (S.H.S.); eunkbang@kist.re.kr (E.K.B.); 3 Department of Medicinal Chemistry, Faculty of Pharmacy, Mansoura University, Mansoura 35516, Egypt; 4 College of Pharmacy, Keimyung University, Daegu 42601, Korea; jihyun96031@naver.com (J.H.K.); seoyho@kmu.ac.kr (Y.H.S.); 5 New Drug Development Center, Daegu-Gyeongbuk Medical Innovation Foundation, Daegu 41061, Korea; leeyuri45@dgmif.re.kr (Y.L.); yujihoon@dgmif.re.kr (J.H.Y.); 6 Division of Bio-Medical Science & Technology, KIST School, Korea University of Science and Technology (UST), Seoul 02792, Korea

**Keywords:** Hsp90 inhibitors, BIIB021 analogs, isoxazole, hydrophobic binding pocket, X-ray crystallography

## Abstract

Inhibition of the molecular chaperone heat shock protein 90 (Hsp90) represents a promising approach for cancer treatment. BIIB021 is a highly potent Hsp90 inhibitor with remarkable anticancer activity; however, its clinical application is limited by lack of potency and response. In this study, we aimed to investigate the impact of replacing the hydrophobic moiety of BIIB021, 4-methoxy-3,5-dimethylpyridine, with various five-membered ring structures on the binding to Hsp90. A focused array of *N*^7^/*N*^9^-substituted purines, featuring aromatic and non-aromatic rings, was designed, considering the size of hydrophobic pocket B in Hsp90 to obtain insights into their binding modes within the ATP binding site of Hsp90 in terms of π–π stacking interactions in pocket B as well as outer α-helix 4 configurations. The target molecules were synthesized and evaluated for their Hsp90α inhibitory activity in cell-free assays. Among the tested compounds, the isoxazole derivatives **6b** and **6c**, and the sole six-membered derivative **14** showed favorable Hsp90α inhibitory activity, with IC_50_ values of 1.76 µM, 0.203 µM, and 1.00 µM, respectively. Furthermore, compound 14 elicited promising anticancer activity against MCF-7, SK-BR-3, and HCT116 cell lines. The X-ray structures of compounds **4b**, **6b**, **6c**, **8**, and **14** bound to the *N*-terminal domain of Hsp90 were determined in order to understand the obtained results and to acquire additional structural insights, which might enable further optimization of BIIB021.

## 1. Introduction

Heat shock protein 90 (Hsp90) is an adenosine triphosphate (ATP)-reliant molecular chaperone that plays a fundamental role in preserving protein homeostasis by regulating the correct folding, intracellular disposition, and activity of numerous client proteins [1,2,3]. A significant number of Hsp90 client proteins are oncoproteins, such as receptor tyrosine kinases; human epidermal growth factor receptor 2 (Her2), epidermal growth factor receptor (EGFR), mesenchymal epithelial transition factor receptor (Met), rapidly accelerated fibrosarcoma-C (c-Raf), cell cycle regulators cyclin dependent kinase 4 (Cdk4), and hypoxia-inducible factor 1α (HIF-1α), which are necessary for the malignant transformation and tumor development [4,5]. Under the inimical environment in cancers such as reduced oxygenation and nutrition deprivation, Hsp90 is generally overexpressed 2–10-fold higher than that in normal cells, and forms activated complexes with its clients in cancer cells [6,7]. Therefore, cancer cells are highly dependent on Hsp90 chaperoning function for their proliferation and survival. Moreover, inhibition of Hsp90 chaperone machinery results in proteasomal degradation of various client oncoproteins that are implicated in multiple signaling pathways in cancers, offering merit of circumventing the common drug resistance of classical chemotherapies [8]. In this regard, Hsp90 inhibition represents a legitimate therapeutic approach for the treatment of cancers [3]. 

**Figure 1:**
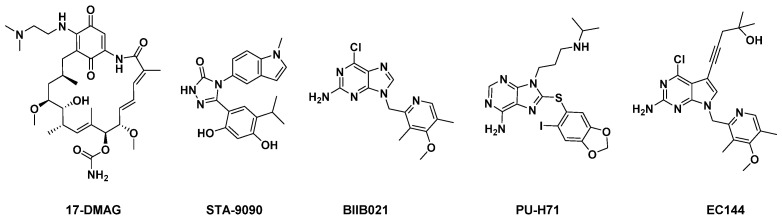
Representative examples of Heat shock protein 90 (Hsp90) inhibitors in clinical trials.

Over the past few years, a plethora of Hsp90 inhibitors endowed with anticancer activity has been developed [9,10,11,12,13,14]. Despite the remarkable clinical efficacies of some Hsp90 inhibitors, they were associated with potential off-target and/or HSP90-relevant toxicities, which hampered their further therapeutic application [15,16,17]. For example, tanespimycin (17-AAG) [18] and alvespimycin (17-DMAG) [19] (Figure 1), the first-generation geldanamycin analogs, have displayed significant hepatotoxicity, which might stem from their quinone structure [20]. Moreover, visual disorders such as night blindness and blurred vision were reported for some chemically diverse second-generation HSP90 inhibitors such as ganetespib (STA-9090) [21,22]. Moreover, the highly potent antitumor purine derivative BIIB021 [23] (Figure 1) showed a high maximum tolerated dose (MTD), but lacked potency and prolonged response, requiring higher doses to achieve biological effects in clinical trials [24]. These clinically reported drawbacks of Hsp90 inhibitors attracted the curiosity of medicinal chemists, leading them to develop new chemical entities in an attempt to achieve comparable potency, improved target selectivity, and better safety profile.

**Figure 2:**
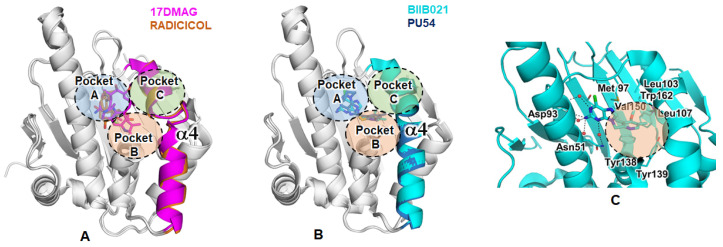
Structure of the *N*-terminal ATP-binding domain of Hsp90 (Hsp90ND) complexed with various inhibitors. The cartoon presentation of Hsp90ND in complex with (**A**) non-purine based inhibitors alvespimycin (17-DMAG; Protein Data Bank (PDB): 1OSF, magenta) and radicicol (PDB: 1BGQ, orange), and (**B**) purine based inhibitors BIIB021 (PDB: 3QDD, cyan) and PU54 (PDB: 3O0I, blue). Inhibitors are shown in the stick model with the helix α4 highlighted in the same color as the inhibitor. The three binding pockets A, B, and C are indicated by circles in blue, orange, and green, respectively. (**C**) The interactions of BIIB021 in the complex structure (PDB code: 3QDD). The dashed line indicates hydrogen bonds.

The majority of investigated HSP90 inhibitors are ATP-competitive, occupying the ATP binding pocket at the *N*-terminal of HSP90 [17]. Guided by the computational analysis of the co-crystal structure/docking investigations of Hsp90 inhibitors, Taldone et al. categorized them on the basis of their interactions with three essential *N*-terminal binding regions: the ATP-binding pocket A, hydrophobic pocket B, and a solvent-exposed exit pocket C [25]. Besides these three major binding pockets, it was reported that the α-helix 4 region (residues 99–125) in the *N*-terminal domain of Hsp90 could show ligand-induced conformational changes that contribute to the Hsp90 selective inhibition (Figure 2A,B). The structurally diverse scaffolds of Hsp90 inhibitors showed distinct ligand-induced conformational changes. While most Hsp90 inhibitors such as 17-DMAG and radicicol adopt the open-loop conformation, both purines BIIB021 and PU-H71 adopted the extended α-helix 4 conformation outside of pocket B [26,27]. These insights might aid in optimizing scaffolds to improve target selectivity, reducing the time and cost required to develop novel inhibitors.

Among the arsenal of Hsp90 inhibitors worthy for further structural exploration, BIIB021, the first orally bioavailable inhibitor, is of special interest. BIIB021 exerted potent anticancer efficacy in the N87 gastric tumor model along with its feasible straightforward synthesis [23]. Furthermore, BIIB021 exhibited a strong antitumor effect against various head and neck squamous cell carcinoma (HNSCC) models, with a remarkable ability to sensitize those HNSCC xenografts to radiotherapy [28]. Moreover, BIIB021 has elicited broad application against cancers with acquired multidrug resistance [29]. The X-ray crystal structure of the Hsp90-BIIB021 complex (PDB code: 3QDD) revealed that the 2-amino and 9-pyridyl moieties of purine BIIB021 bind to adenine binding pocket A and the hydrophobic pocket B, respectively (Figure 2C) [23]. The pyridine ring in BIIB021 was involved in multiple hydrophobic interactions including π–π stacking with the phenyl ring of Phe138, π–σ stacking with isopropyl of Leu107, as well as π–edge contacts with the ring structures of Tyr139 and Trp162. Guided by the structural data, the 7-deazapurine derivative EC144 was developed, where the N7 of BIIB021 was replaced with a carbon atom substituted with 2-methylpent-4-yn-2-ol [26], while the hydrophobic binding moiety, 4-methoxy-3,5-dimethylpyridine, was conserved. Such a study provided EC144 with approximately 20-fold higher efficacy than BIIB021 in mice [26]. However, the impact of structural variation of the 9-pyridine moiety with simple five-membered heterocyclic structures has not been investigated.

**Figure 3:**
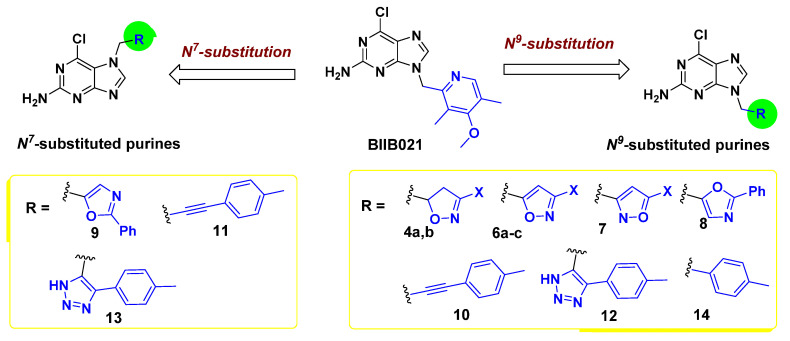
Proposed structural modification of BIIB021.

To fulfill this structure–activity relationship (SAR) gap, and as a continuation of our efforts to identify potent anticancer chemotherapeutics [11,30,31,32,33,34,35], we were motivated to synthesize a focused library of *N*^7^/*N*^9^-substituted purines. In this study, we focused on the hydrophobic binding pocket B, a newly formed binding pocket of Hsp90ND created by the conformational change. In this line, we conducted a structural exploration study to highlight the tolerability of changing the 4-methoxy-3,5-dimethylpyridine of BIIB021 with simpler aromatic and non-aromatic five-ring structures, such as isoxazoline, isoxazole, oxazole, and triazole featuring bulky substituents (ethyl carboxylate, *ter*-butyl, isopropyl, phenyl) (Figure 3). We hypothesized that such designed molecules possessing nitrogen and/or oxygen atoms might offer binding mode(s) that are different from that of BIIB021, a variable degree of π–π stacking interactions with pocket B, and their terminal bulky moieties might result in a different configuration of the outer α-helix 4. The target molecules were synthesized and evaluated for their Hsp90α inhibitory activity in cell-free assays. Moreover, the X-ray crystal structures of several members of our target compounds with Hsp90 were determined to obtain reliable structural insights that would lead to a better understanding of the compound’s binding mode, therefore paving the way for proper further optimization.

## 2. Results and Discussion

### 2.1. Chemical Synthesis

**Scheme 1:**
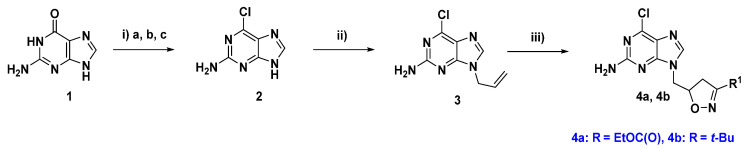
Synthetic pathway for compounds **4a**,**b**. Reagent and reaction conditions: (**i**) (**a**) POCl_3_, ClCH_2_CH_2_Cl, dimethylformamide (DMF), 80 °C, 80%; (**b**) CH_3_CO_2_H, 70 °C; (**c**) NaOH, HCl, room temperature (rt), 80% in three steps; (**ii**) NaH, allyl bromide, CH_3_CN, rt, 50%; (**iii**) appropriate oxime, Triethylamine (TEA), Dichloromethane (DCM), 0 °C to rt, 34% (**4a**), 35% (**4b**).

The novel target compounds were synthesized from 6-chloro-purin-2-amine **2**, which was generated via chlorination of the imine-protected nucleobase guanine **1** using dimethylformamide (DMF) [36] (Scheme 1). *N*-allylation of **2** using allyl bromide provided the *N^9^*-allyl regioisomers **3** in 50% yield. 1,3-Dipolar cycloaddition [37] of the allyl derivative **3** with the appropriate oxime generated the corresponding isoxazolines **4a** and **4b**.

**Scheme 2:**
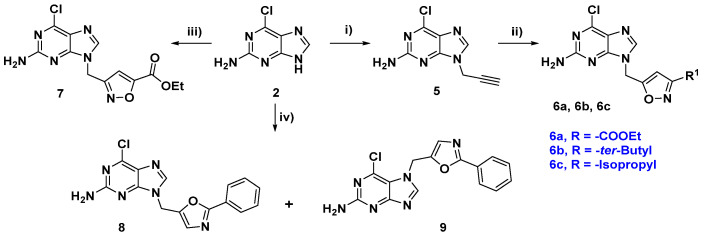
Synthetic pathway for compounds **6a**–**c** and **7**–**9**. Reagent and reaction conditions: (**i**) HC≡CCH_2_Br, K_2_CO_3_, DMF, rt, 49%; (**ii**) appropriate oxime, TEA, Tetrahydrofuran (THF), 0 °C to rt, 12% (**6a**), 90% (**6b**), 35% (**6c**); (**iii**) ethyl 3-(chloromethyl)isoxazole-5-carboxylate, *ter*-BuONa, DMF, rt, 63%; (**iv**) 5-(bromomethyl)-2-phenyloxazole, K_2_CO_3_, DMF, rt, 25% (**8**), 64% (**9**).

On the other hand, the isoxazole derivatives **6a**–**c** were obtained from the *N*^9^-methylene propargyl purine **5** through the same cycloaddition with propionitrile oxides, as shown in Scheme 2 [38]. Treatment of compound **2** with the reverse 3,5-substituted isoxazole in DMF using sodium *tert*-butoxide furnished the corresponding *N*^9^-substituted purine **7** in a 63% yield. Treatment of 5-(bromomethyl)-2-phenyloxazole with compound **2** in K_2_CO_3_/DMF produced the *N*^9^- and *N*^7^-isomers 8 and 9 in 25% and 64% yields (1:2.5), respectively. 

**Scheme 3:**
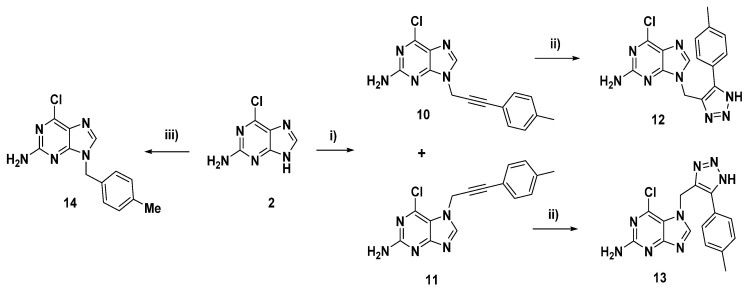
Synthetic pathway for compounds **10**–**14**. Reagent and reaction conditions: (**i**) 1-(3-chloroprop-1-yn-1-yl)-4-methylbenzene, NaI, K_2_CO_3_, DMF, rt, 10% (**10**), 59% (**11**); (**ii**) I_2_, NaN_3_, DMF, 120 °C, 54% (**12**), 43% (**13**); (**iii**) 4-methylbenzyl chloride, K_2_CO_3_, DMF, rt, 73%.

Referring to Scheme 3, treatment of **2** with 1-(3-chloroprop-1-yn-1-yl)-4-methylbenzene afforded the *N*^9^ and *N*^7^-regioisomers **10** and **11** in 10% and 59% yields (1:6), respectively. Click reactions of **10** and **11** with sodium azide produced the corresponding *p*-tolyl-substituted 1,2,3-triazoles **12** and **13**, with moderate yields. The 4-methylbenzyl-substituted purine congener **14** was achieved in 73% yield through *N*-benzylation of **2** with 4-methylbenzyl chloride in DMF using potassium carbonate.

### 2.2. Biological Evaluation 

#### 2.2.1. Assessment of Hsp90α Binding Affinity Using FP Assay

First, the fluorescence polarization (FP) assay, a well-validated method for high-throughput screening [39], was adopted for determining the binding affinities. The FP assay is based on competition for the ATP-binding pocket of Hsp90α by a test compound and a fluorescently labeled geldanamycin (GM) probe such as the synthesized green or red boron-dipyrromethene (BODIPY)-GM [40] and fluorescein isothiocyanate labeled geldanamycin (FITC-GM) [41,42]. All compounds were tested in a duplicate mode against *N*-terminal human Hsp90α with BODIPY-GM to calculate their inhibitory constant Ki after 5 h treatment, which was found to be >1.9 μM. Next, we selected an array of compounds for measuring their binding affinity to Hsp90α using the FITC-GM probe at Reaction Biology Corporation (RBC, Malvern, PA, USA) [43]. BIIB021 and 17-AAG were used as references, and the results are summarized in Table 1. The obtained data revealed that the five-membered ring derivatives exhibited rather weak binding affinities, presumably caused by the lower π-stacking interaction force of the five-membered ring compared to the six-membered ring. The 3-*tert*-butyl isoxazole **6b** showed the best binding affinity (95.8% inhibition) among the five-membered ring members. Moreover, **6b** elicited a 2.7-fold superior potency to the non-aromatic isoxazoline congener **4b** (35.4% inhibition). Furthermore, compound **6b** showed an IC_50_ value of 1.76 μM. Replacing the 3-*tert*-butyl moiety of isoxazole with less bulky isopropyl showed comparable inhibitory affinity (**6c**, 77.3% inhibition). However, this compound **6c** displayed an affinity twofold higher than **4b** and exerted a considerable Hsp90α inhibitory effect with an IC_50_ value of 0.203 μM. Inspection of the more extended propionate ester derivatives such as isoxazoline **4a** and isoxazole **6a** disclosed their low potency (less than 15% inhibition). The phenyl oxazole **8**, featuring a lower rotational group, displayed an affinity (48.7% inhibition) twofold higher than the ester derivatives of isoxazole. Compound **9**, the positional isomer of compound **8**, as well as the *p*-tolyl acetylene **11** and triazole derivatives **12** and **13** displayed a fluorescence background that interfered with the assay. Interestingly, the sole six-membered ring derivative **14**, bearing 4-methylbenzyl moiety, displayed a favorable binding affinity with 95.4% inhibition at the tested dose of 10 μM. Further assay of **14** revealed its promising IC_50_ value of 1.00 μM. 

#### 2.2.2. In Vitro Antiproliferative Activity

The in vitro anticancer activity of compounds **4b**, **6b**, **6c**, **8**, and **14** was evaluated against a panel of three human cancer cells expressing high levels of Hsp90-related client proteins: Her-2 dependent SK-BR-3 breast cancer, estrogen receptor positive (ER+) MCF-7 breast cancer, and EGFR-reliant colon (HCT116) cancer cell line, using BIIB021 and 17-AAG as references. The growth inhibitory effects of the test compounds, at 10 µM dose, were determined after 72 h incubation using the 3-(4,5-dimethylthiazol-2-yl)-2,5-diphenyl tetrazolium bromide (MTT) assay. As revealed from the data listed in Table 2, most of the examined compounds exerted modest to moderate cytostatic activity over the tested cancer cell lines. Compound **4b** showed unpretentious antiproliferative activity against all examined cells, with growth inhibition (GI) less than 30%. Such modest cellular outcomes of those purine derivatives may be attributed to their lower lipophilic characters, which in turn restrict their cellular permeability into cancer cells. While comparing the activity of 3-substituted isoxazole **6b** and **6c**, we observed that the most hydrophobic isoxazole **6b**, with *tert*-butyl moiety, showed reasonable activity against SK-BR-3 breast cancer (GI = 45.76%). The most active member in Hsp90α assay **6c** elicited modest anticancer activity (SK-BR-3; GI = 33.17%, MCF-7; GI = 11.92%). The 2-phenyloxazole-substituted purine **8** exerted reasonable antiproliferative activity, with GI values of 33.27% and 37.46% against MCF-7 and SK-BR-3 cells, respectively. Interestingly, compound **14** showed good cellular activity against the tested cell lines, particularly the SK-BR-3 (GI = 60.93%). In view of GI_50_ values, the phenyloxazole **8** showed a GI_50_ value of 22.54 ± 0.70 µM towards SK-BR-3. The *p*-tolyl derivatives **14** exhibited equipotent activity against the breast cancer cells MCF-7 and SK-BR-3, with GI_50_ values of 16.22 ± 0.71 µM and 15.24 ± 2.08 µM, respectively. However, all tested derivatives were less potent than the reference compounds BIIB021 and 17-AAG. 

### 2.3. Biochemical, Cellular, and Structural Evaluation

#### 2.3.1. Assessment of *m*Hsp90ND Binding Affinity Using ITC

**Figure 4:**
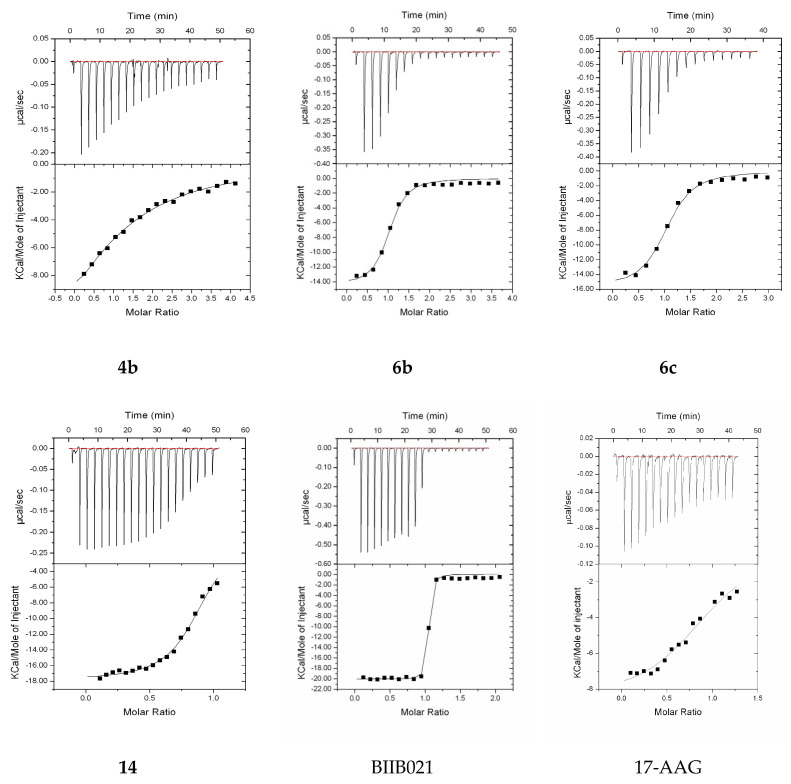
Isothermal titration calorimetry (ITC) data for the binding of the compounds. Injection of the compounds **4b**, **6b**, **6c**, **14**, BIIB021, and 17-AAG to *m*Hsp90ND. The top panel shows the heat effects associated with the injection, while the bottom panel shows the binding isothermal corresponding to the data in the top panel and the best-fitted curve with a one-site binding model.

The binding affinity of the compounds to the *N*-terminal domain of Hsp90α was measured using the isothermal titration calorimetry (ITC). Some of the tested compounds were not as soluble in the buffer solution, but we were able to analyze the binding affinities for compounds **4b**, **6b**, **6c**, and **14**. The results are depicted in Figure 4 and Table 3. They all showed a 1:1 molar binding to the target protein, and the *K*_D_ value for 17-AAG is comparable to the value reported earlier [44]. The five-membered ring isoxazole derivative **6b**, possessing a *tert*-butyl group, displayed a good binding affinity (*K*_D_ = 0.44 µM) as well as the isoxazole **6c**, featuring an isopropyl moiety with a comparable binding affinity (*K*_D_ = 0.68 µM). In contrast, compound **4b**, the isoxazoline analog of **6b**, manifested only a modest activity (*K*_D_ = 15.6 µM). Interestingly, 4-methylphenyl compound **14**, the only six-membered ring derivative, showed the best affinity (*K*_D_ = 0.41 µM), similar to isoxazoles **6b** and **6c**.

#### 2.3.2. Colorimetric Determination of ATPase Activity of Hsp90

**Figure 5:**
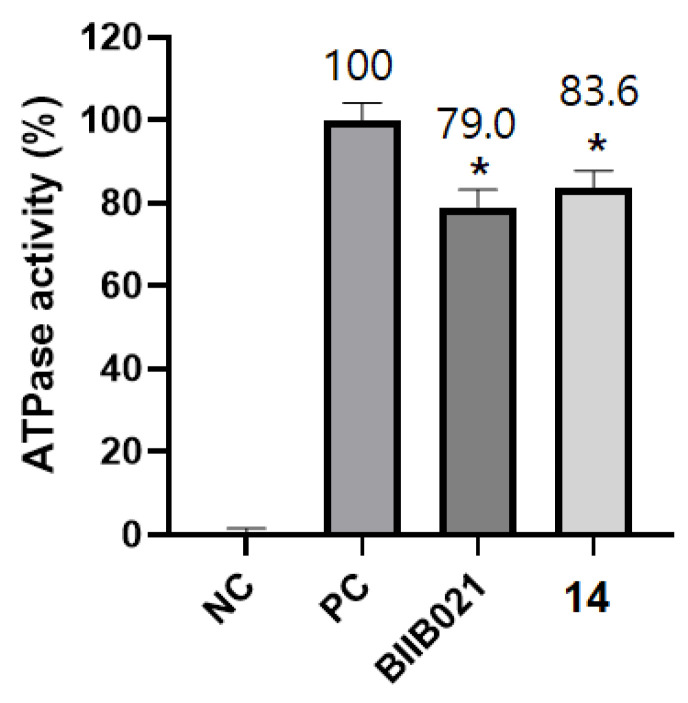
Inhibition of ATPase activity of Hsp90α. ATPase activity was measured in the presence of BIIB021 and compound **14** (50 µM) compared to positive control with Hsp90α. Data are the mean of experiments performed in duplicate and were analyzed by *t*-test (positive control (PC) vs. testing compounds). The error bar represents the standard deviation (SD), while * indicates significance at *p* ≤ 0.05. NC, negative control, PC, positive control.

Since the intrinsic ATPase activity of Hsp90 is pivotal for its chaperone function and as a part of our study, we tested the tendency of compound **14,** which exhibited favorable binding affinity with Hsp90, to inhibit Hsp90 by measuring the ATPase activity of Hsp90 along with BIIB021 as a known inhibitor. As revealed from the data (Figure 5), the ATPase activity of Hsp90 was decreased upon incubation with compound **14** in a pattern comparable to that observed with BIIB021. This finding might emphasize that the binding of compound **14** to Hsp90 would result in inhibition of its ATPase activity. 

#### 2.3.3. Western Blot Analysis of HSP90 Key Client Protein Expression and Activation

**Figure 6:**
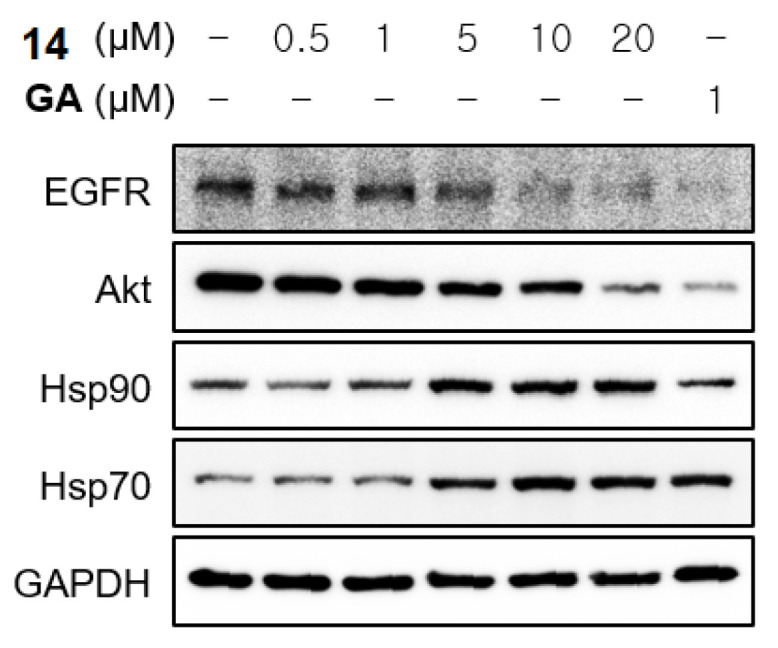
Effect of compound **14** on cellular biomarkers of HSP90 inhibition in MCF7 cells. MCF7 cells were treated with the indicated concentrations of compound **14** for 24 h and the expression levels of epidermal growth factor receptor (EGFR), protein kinase B (Akt/PKB), Hsp90, Hsp70, and glyceraldehyde-3-phosphate dehydrogenase (GAPDH) were analyzed by Western blot. Geldanamycin (GA) was used as a positive control.

Furthermore, the effect of compound **14** on cellular biomarkers of the HSP90 inhibition was evaluated to obtain insights about the molecular mechanisms triggering its anticancer activity. MCF7 cells were incubated with various doses of compound **14** (0.5, 1, 5, 10, and 20 μM) for 24 h, and the expression levels of HSP90 client proteins (EGFR, Akt, and Cdk4), Hsp90, HSP70, and glyceraldehyde-3-phosphate dehydrogenase (GAPDH) were determined by Western blot analysis (Figure 6).

Compound **14** displayed the characteristic signature of Hsp90 inhibitors, where it induced proteasomal degradation of HSP90 oncogenic client proteins (EGFR and Akt) and upregulated the expression level of Hsp70 in a dose-dependent manner, while the levels of GAPDH remained unchanged. The expression levels of EGFR and Akt were remarkably decreased upon the treatment of MCF7 cells with 10 μM and 20 μM of compound **14**, respectively. On the basis of these outcomes, it could be suggested that compound **14** can disrupt the HSP90 chaperoning machinery in MCF7 cells. 

#### 2.3.4. Structural Analysis of the Complexes Using X-ray Crystallography

To understand the binding of these compounds at the atomic level, we determined the crystal structures of compounds **4b**, **6b**, **6c**, **8**, and **14** complexed to the *N*-terminal domain of mouse Hsp90 at the resolutions ranging from 1.33 to 1.75 Å. The final statistics on the data collection and refinement are summarized in Table 4. 

**Figure 7:**
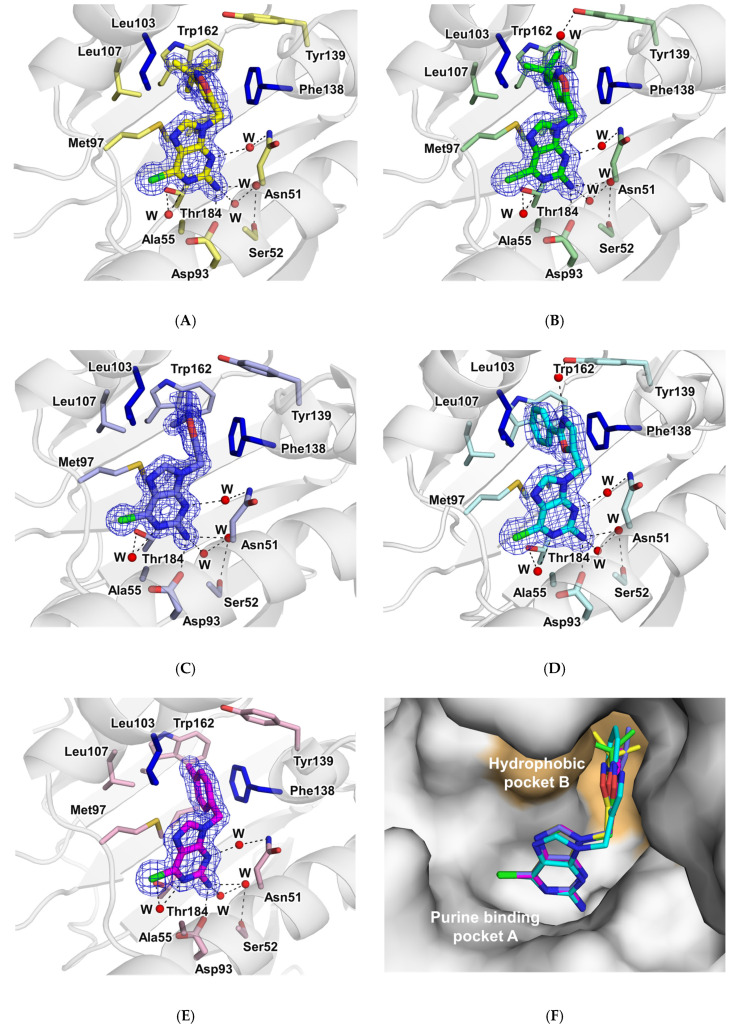
Crystal structures of the *m*Hsp90ND compound complexes. (**A**–**E**) Electron density map (*2Fo-Fc* map contoured at 1.5 σ level) for compounds **4b** (yellow), **6b** (green), **6c** (violet), **8** (cyan), and **14** (pink), respectively, in the complex structures. The dashed line indicates hydrogen bond. (**F**) Superposition of the compounds **4b**, **6b**, **6c**, **8**, and **14** shown in the stick model in the same color as in (**A**–**E**), with the surface of *m*Hsp90ND with the hydrophobic pocket B highlighted in orange.

**Figure 8:**
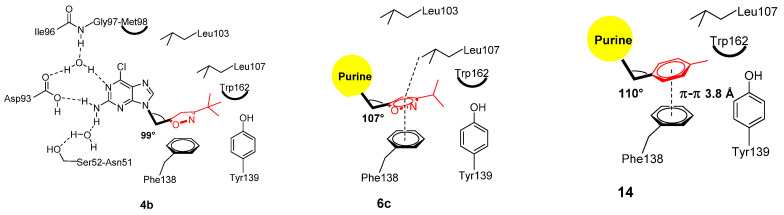
2D binding modes of compounds **4b**, **6c**, and **14** in the *N*-terminal active site of Hsp90. The yellow circle refers to the purine ring.

The complex structures confirmed that compounds **4b**, **6b**, **6c**, **8**, and **14** are tightly bound to the ATP-binding site of Hsp90, with all atoms well defined in electron density maps (Figure 7A–E). In all cases, the electron density map gave unambiguous positioning of the compounds as well as the protein atoms near the ligands and water molecules. Compounds **4b**, **6b**, **6c, 8,** and **14** all showed an L-shape conformation, occupying the two binding pockets as expected. The purine ring is located above the β-sheet tightly bound in the binding A pocket, making extensive hydrophobic interactions with protein atoms. Moreover, it makes tight hydrogen bonds to Asp93 and two water molecules that are involved in an intricate hydrogen bond network with protein atoms. This interaction was found in all five structures reported here. The isoxazoline moiety of **4b**, isoxazoles of **6b** and **6c**, and phenyloxazole of **8** bound to the hydrophobic pocket B are oriented in a parallel direction to the phenyl ring of Phe138, isopropyl of Leu107, and perpendicularly to the rings of Tyr139 and Trp162, as seen in the figure. Figure 7F illustrates the superposition of the five investigated compounds. Contrastingly to the purine ring, these aromatic rings showed certain variations in their orientation, with **4b** and **8** in particular displaying significant differences. The ITC data (Table 3) of **4b** suggested a distinct behavior from the rest. Interestingly, the angle between the purine ring and the isoxazoline in the case of **4b** was only 99°, while it ranged from 107° to 110° in others (Figure 8). The non-planar isoxazoline ring of **4b** with a bulky substituent at position 3 made the angle between methylene substituents constrained in the hydrophobic pocket B by conserving π–π stacking interaction with phenyl ring of Phe138 without breaking outer helix 4. In the case of **8**, the methylene atom connecting purine and phenyloxazole was moved outwards into the solvent by 0.5 Å compared to what was seen in others. However, we were not able to obtain the ITC data on **8** due to its low solubility. The phenyl ring in phenyloxazole of **8** is too bulky to slide sufficiently into the inside of the pocket B. In all five structures, the conformation of the protein atoms in the B pocket remained almost the same, suggesting that the B pocket is highly selective in terms of the tight binding and the rigid space.

Overall, these interactions are quite similar to that of BIIB021, as expected, i.e., the purine made almost identical interactions while the pyridine ring was oriented in a parallel direction to the phenyl ring of Phe138 (π–π stacking distance of 3.7 Å) and isopropyl of Leu107 (π–σ stacking distance of 3.9 Å) and perpendicularly to the rings of Tyr139 and Trp162 (Figure 2C). In contrast, the crystal structures here pointed out that the isoxazoline derivative **4b**, isoxazoles **6b** and **6c**, and phenyloxazole **8** showed less π stacking interactions, in a variable degree, with Phe138 and Leu107, due to either having lower aromaticity than the benzene ring (**6b**, **6c**, and **8**) or even lacking aromaticity entirely (**4b**).

Thus, these five-membered ring-featuring purines had a relatively less stable conformation than BIIB021. Isoxazoline **4b** demonstrated a smaller angle (99°) of the methylene bond between the purine ring and the isoxazoline ring. Moreover, our structural data indicated that the various bulky substituents on heterocyclic rings did not alter the conservation of outer α-helix 4. Interestingly, it was found that the sole synthesized six-membered member **14** showed a similar binding pattern to that of BIIB021, where the *p*-tolyl moiety in **14** was packed against the side chain of Leu107 through π–σ stacking, in addition to the significant face-to-face stacking with Phe138 residue (π–π stacking distance of 3.8 Å) in the hydrophobic B pocket. Moreover, the tolyl structural domain was in a perpendicular direction to the rings of hydrophobic residues Tyr139 and Trp162, which further contributed to the stabilization of **14** within the ATP-binding pocket of *m*Hsp90ND. In light of these results, it could be concluded that the inhibitor’s conformation stability inside the active site pocket would influence its binding affinity and consequently the biological activity.

## 3. Materials and Methods 

### 3.1. Chemistry

#### 3.1.1. General Methods

All solvents and reagents were purchased from commercial suppliers and were used directly without purification. The reactions, applying the standard Schlenk techniques, were monitored using the thin layer chromatography (TLC) plate (Merck, silica gel 60 F254). The final compounds were purified by column chromatography using silica gel (Merck, 230–400 mesh) and the eluent solvents as indicated. The purity of all final compounds was >95%. ^1^H and ^13^C NMR charts were recorded on a Bruker Avance 400 or 300 MHz spectrometers (Bruker, Rheinstetten, Germany), using the appropriate deuterated NMR solvent. Chemical shifts (δ) are given in parts per million (ppm) upfield from the internal standard tetramethylsilane (TMS). The singlet, doublet, triplet, and multiplet peaks were indicated as s, d, t, and m, respectively, and the coupling constants (J) are reported in hertz (Hz). High-resolution mass spectra (HRMS) were recorded on either JMS 700 (Jeol, Tokyo, Japan) or Waters Acquity UPLC/Synapt G2 QTOF MS mass spectrometer (Waters, MA, USA).

#### 3.1.2. Synthesis and Structural Characterization

***9-Allyl-6-chloro-9H-purin-2-amine (3)***: To a suspension of sodium hydride (75 mg, 1.97 mmol) in CH_3_CN (2.0 mL), we added 2-amino-6-chloropurine (300 mg, 1.79 mmol) at room temperature. After 1 h, allyl bromide (0.28 mL, 3.22 mmol) was slowly added to the solution. The reaction mixture was stirred at the same temperature for 48 h, and concentrated under reduced pressure. The residue was purified by column chromatography with CH_2_Cl_2_/MeOH (40/1, *v*/*v*) to afford compound **3** (187 mg, 50% yield); ^1^H NMR (400 MHz, DMSO-*d*_6_) *δ* 8.19 (s, 1H), 6.92 (s, 2H), 6.07–5.98 (m, 1H), 5.18 (d, *J* = 10.3 Hz, 1H), 4.98 (d, *J* = 17.1 Hz, 1H), 4.68 (d, *J* = 5.0 Hz, 2H); ^13^C NMR (100 MHz, DMSO-*d*_6_) *δ* 160.3, 154.4, 149.9, 143.6, 133.4, 123.7, 117.8, 45.4.

***Ethyl 5-((2-amino-6-chloro-9H-purin-9-yl)methyl)-4,5-dihydroisoxazole-3-carboxylate (4a)***: To a suspension of compound **3** (100 mg, 0.48 mmol) in CH_2_Cl_2_ (8 mL), we added ethyl (*Z*)-2-chloro-2-(hydroxyimino)acetate (108 mg, 0.72 mmol) and trimethylamine (TEA) (0.77 mL, 5.51 mmol) in CH_2_Cl_2_ (2 mL) at 0 °C. The reaction was stirred at room temperature for 24 h, and was concentrated under reduced pressure. The residue was purified by column chromatography with CH_2_Cl_2_/MeOH (40/1, *v*/*v*) to provide the title product **9a** (52 mg, 34% yield): ^1^H NMR (300 MHz, DMSO-*d*_6_) δ 7.91 (s, 1H), 4.43–4.33 (m, 4H), 3.91 (s, 1H), 3.43–3.32 (m, 1H), 3.11–3.03 (m, 1H), 1.39 (t, *J* = 7.1 Hz, 3H); ^13^C NMR (75 MHz, DMSO-*d*_6_) δ 160.0, 159.2, 153.9, 151.8, 142.8, 125.0, 80.8, 62.5, 45.8, 36.5, 14.1.

***9-((3-(tert-Butyl)-4,5-dihydroisoxazol-5-yl)methyl)-6-chloro-9H-purin-2-amine (4b)***: To a solution of (*E*)-pivalaldehyde oxime (58 mg, 0.57 mmol) in tetrahydrofuran (THF) (2 mL), we added *N*-chlorosuccinimide (96 mg, 0.72 mmol) at 0 °C. After 2 h, the mixture solution was dropwised to a reaction mixture of compound **5** (100 mg, 0.48 mmol) and TEA (0.10 mL, 0.72 mmol) in THF (2 mL). The reaction was stirred at room temperature for 24 h, and was concentrated under reduced pressure. The residue was purified by column chromatography with CH_2_Cl_2_/MeOH (40/1, *v*/*v*) to afford the title product **4b** (49 mg, 35% yield): mp 198.3−200.8 °C; ^1^H NMR (300 MHz, DMSO-*d*_6_) *δ* 8.03 (s, 1H), 6.93 (s, 2H), 4.93–4.88 (m, 1H), 4.15 (s, 2H), 3.20–3.10 (m, 1H), 2.94–2.87 (m, 1H), 0.96 (s, 9H); ^13^C NMR (75 MHz, DMSO-*d*_6_) *δ* 165.9, 160.3, 154.8, 149.9, 144.1, 123.5, 77.4, 46.7, 37.3, 32.9, 28.1; HRMS electrospray ionization-time-of-flight (ESI-TOF) *m*/*z* calcd for C_13_H_17_ClN_6_O [M-H^+^] 308.1152, found 307.1074.

***6-Chloro-9-(prop-2-yn-1-yl)-9H-purin-2-amine (5)*** [45]: A suspension of 2-amino-6-chloropurine **2** (1.0 g, 5.90 mmol), propargyl bromide (0.71 mL, 5.90 mmol), and K_2_CO_3_ (2.45 g, 1.77 mmol) in DMF (7 mL) was stirred at room temperature (rt) for 10 h. The reaction solvent was evaporated under reduced pressure, and the residue was purified by column chromatography with dichloromethane/methanol (50/1, *v*/*v*) to afford the title compound **5** (604 mg, 49% yield): (300 MHz, DMSO-*d*_6_) *δ* 8.19 (s, 1H), 6.92 (s, 2H), 6.07–5.98 (m, 1H), 5.18 (d, *J* = 10.3 Hz, 1H), 4.98 (d, *J* = 17.1 Hz, 1H), 4.68 (d, *J* = 5.0 Hz, 2H); ^13^C NMR (75 MHz, DMSO-*d*_6_) *δ* 160.3, 154.4, 149.9, 143.6, 133.4, 123.7, 117.8, 45.4.

***Ethyl 5-((2-amino-6-chloro-9H-purin-9-yl)methyl)isoxazole-3-carboxylate (6a)***: To a suspension of compound **5** (20 mg, 0.13 mmol) and ethyl (*Z*)-2-chloro-2-(hydroxyimino)acetate (40 mg, 0.19 mmol) in THF (4 mL), we added TEA (0.27 mL, 0.13 mmol) dropwise in THF (2 mL) at 0 °C. The reaction was stirred at room temperature for 24 h, and was concentrated under reduced pressure. The residue was purified by column chromatography with CH_2_Cl_2_/MeOH (40/1, *v*/*v*) to provide the title product (5 mg, 12% yield): ^1^H NMR (400 MHz, CDCl_3_) *δ* 7.88 (s, 1H), 6.65 (s, 1H), 5.44 (s, 2H), 5.17 (bs, 2H), 4.45 (q, *J* = 7.9 Hz, 2H), 1.40 (t, *J* = 7.1 Hz, 3H); ^13^C NMR (75 MHz, CDCl_3_) *δ* 167.3, 159.4, 159.3, 156.8, 153.4, 151.9, 141.2, 124.9, 104.3, 62.5, 38.4, 14.1.

***General procedure for synthesis of compounds 6b and 6c***: To a solution of (*E*)-pivalaldehyde oxime or (*E*)-isobutyraldehyde oxime (0.48 mmol) in THF (2 mL), we added *N*-chlorosuccinimide (96 mg, 0.72 mmol) at 0 °C. After 2 h, the resulting solution was added dropwise to a reaction mixture of compound **5** (100 mg, 0.48 mmol) and TEA (0.10 mL, 0.72 mmol) in THF (2 mL). The reaction was stirred at room temperature for 24 h, and was concentrated under reduced pressure. The residue was purified by column chromatography with CH_2_Cl_2_/MeOH (40/1, *v*/*v*) to afford the desired product.

***9-((3-(tert-Butyl)isoxazol-5-yl)methyl)-6-chloro-9H-purin-2-amine (6b)***: Yield 90%: ^1^H NMR (300 MHz, DMSO-*d*_6_) *δ* 8.23 (s, 1H), 7.01 (s, 2H), 6.40 (s, 1H), 5.45 (s, 2H), 1.21 (s, 9H); ^13^C NMR (75 MHz, DMSO-*d*_6_) *δ* 172.4, 167.0, 160.5, 154.4, 150.1, 143.3, 123.5, 101.3, 32.2, 29.5; HRMS (ESI-TOF) *m*/*z* calcd for C_13_H_15_ClN_6_O [M + Na^+^] 306.0996, found 329.0894. 

***6-Chloro-9-((3-isopropylisoxazol-5-yl)methyl)-9H-purin-2-amine (6c)***: Yield 35%: ^1^H NMR (300 MHz, CDCl_3_) *δ* 7.88 (s, 1H), 6.07 (s, 1H), 5.42 (bs, 2H), 5.35 (s, 2H), 3.07−2.98 (m, 1H), 1.25 (d, *J* = 6.8 Hz, 6H); ^13^C NMR (75 MHz, CDCl_3_) *δ* 169.7, 165.0, 159.4, 153.5, 151.7, 141.6, 124.9, 101.7, 38.5, 26.5, 21.6; HRMS (FAB+) *m*/*z* calcd for C_12_H_14_ClN_6_O [M+H]^+^: 293.0917, found: 293.0920.

***Ethyl 3-((2-amino-6-chloro-9H-purin-9-yl)methyl)isoxazole-5-carboxylate (7)***: A suspension of compound **2** (100 mg, 0.59 mmol), ethyl 3-(chloromethyl)isoxazole-5-carboxylate (125 mg, 0.66 mmol), and sodium *tert*-butoxide (57 mg, 0.59 mmol) in DMF (2 mL) was stirred at rt for 9 h. The reaction solvent was evaporated under reduced pressure, and the residue was purified by column chromatography with dichloromethane/methanol (40/1, *v*/*v*) to afford the title compound **16** (120 mg, 63% yield): ^1^H NMR (300 MHz, DMSO-*d*_6_) *δ* 8.25 (s, 1H), 7.28 (s, 1H), 7.01 (s, 2H), 5.48 (s, 2H), 4.33 (q, *J* = 7.1 Hz, 2H), 1.28 (t, *J* = 7.1 Hz, 3H); ^13^C NMR (75 MHz, DMSO-*d*_6_) *δ* 161.4, 160.8, 160.4, 156.4, 154.5, 150.0, 143.5, 123.6, 109.4, 62.6, 14.3.

***Synthesis of compounds 8 and 9***: A suspension of compound **2** (161 mg, 0.95 mmol), 5-(bromomethyl)-2-phenyloxazole (227 mg, 0.95 mmol), and K_2_CO_3_ (394 mg, 2.85 mmol) in DMF (2 mL) was stirred at rt for 11 h. The reaction solvent was evaporated under reduced pressure, and the residue was purified by column chromatography with dichloromethane/methanol (30/1, *v*/*v*) to afford compounds **8** (78 mg, 25% yield) and **9** (198 mg, 64% yield).

***6-Chloro-9-((2-phenyloxazol-5-yl)methyl)-7H-purin-2-amine (8)***: ^1^H NMR (300 MHz, DMSO-*d*_6_) *δ* 8.27 (s, 1H), 7.94–7.91 (m, 2H), 7.52–7.50 (m, 3H), 7.32 (s, 1H), 7.03 (s, 2H), 5.47 (s, 2H); ^13^C NMR (75 MHz, DMSO-*d*_6_) *δ* 161.4, 160.4, 154.3, 150.0, 147.3, 143.3, 131.3, 129.6, 127.4, 127.0, 126.4, 123.6, 37.9; HRMS (FAB+) *m*/*z* calcd for C_15_H_6c_lN_6_O [M+H]^+^: 327.0761, found: 327.0759.

***6-Chloro-7-((2-phenyloxazol-5-yl)methyl)-7H-purin-2-amine (9)***: ^1^H NMR (300 MHz, DMSO-*d*_6_) *δ* 8.57 (s, 1H), 7.92–7.89 (m, 2H), 7.52–7.50 (m, 3H), 7.37 (s, 1H), 6.70 (s, 2H), 5.73 (s, 2H); ^13^C NMR (75 MHz, DMSO-*d*_6_) *δ* 164.7, 161.4, 160.6, 150.1, 147.8, 142.9, 131.3, 129.7, 127.3, 127.0, 126.3, 115.1, 41.2.

***Synthesis of compounds 10 and 11:*** A suspension of compound **2** (113 mg, 0.67 mmol), 1-(3-chloroprop-1-yn-1-yl)-4-methylbenzene (110 mg, 0.67 mmol), sodium iodide (0.01 mg, 0.08 mmol), and K_2_CO_3_ (277 mg, 2.00 mmol) in DMF (3.3 mL) was stirred at rt for 12 h. The reaction solvent was evaporated under reduced pressure, and the residue was purified by column chromatography with dichloromethane/methanol (30/1, *v*/*v*) to afford both compounds **10** (42 mg, 10% yield) and **11** (261 mg, 59% yield).

***6-Chloro-9-(3-(p-tolyl)prop-2-yn-1-yl)-9H-purin-2-amine (10):***^1^H NMR (300 MHz, DMSO-*d*_6_) *δ* 8.26 (s, 1H), 7.33 (d, *J* = 8.0 Hz, 2H), 7.18 (d, *J* = 6.7 Hz, 2H), 7.04 (s, 2H), 5.18 (s, 2H), 2.30 (s, 3H); ^13^C NMR (75 MHz, DMSO-*d*_6_) *δ* 160.4, 154.1, 150.0, 142.9, 139.3, 131.9, 129.8, 123.6, 118.8, 84.8, 83.3, 33.6, 21.5

***6-Chloro-7-(3-(p-tolyl)prop-2-yn-1-yl)-7H-purin-2-amine (11):***^1^H NMR (300 MHz, DMSO-*d*_6_) *δ* 8.49 (s, 1H), 7.33 (d, *J* = 8.0 Hz, 2H), 7.18 (d, *J* = 7.9 Hz, 2H), 6.69 (s, 2H), 5.42 (s, 2H), 2.30 (s, 3H); ^13^C NMR (75 MHz, DMSO-*d*_6_) *δ* 164.8, 160.7, 149.4, 143.0, 139.4, 131.9, 129.8, 118.8, 115.2, 85.8, 83.8, 37.3, 21.5.

***General procedure for synthesis of compounds 12 and 13:*** A solution of **10** or **11** (261 mg, 0.88 mmol), iodine (4.5 mg, 0.02 mmol), and sodium azide (114 mg, 1.76 mmol) in DMF (2.2 mL) was stirred at 120 °C for 9 h. The reaction solvent was evaporated under reduced pressure, and the residue was purified by column chromatography with dichloromethane/methanol (50/1, *v*/*v*) to afford the title compounds.

***6-Chloro-9-((5-(p-tolyl)-1H-1,2,3-triazol-4-yl)methyl)-9H-purin-2-amine (12)***: Yield 54%: ^1^H NMR (300 MHz, DMSO-*d*_6_) *δ* 8.51 (bs, 2H), 8.30 (s, 1H), 7.33 (d, *J* = 8.0 Hz, 1H), 7.18 (d, *J* = 7.7 Hz, 1H), 5.31 (s, 2H), 2.29 (s, 3H); ^13^C NMR (75 MHz, DMSO-*d*_6_) *δ* 146.6, 144.9, 144.4, 140.0, 139.4, 131.9, 129.8, 118.8, 112.2, 84.9, 83.5, 34.2, 21.5.

***6-Chloro-7-((5-(p-tolyl)-1H-1,2,3-triazol-4-yl)methyl)-7H-purin-2-amine (13)***: Yield 43%: ^1^H NMR (300 MHz, DMSO-*d*_6_) *δ* 8.45 (s, 1H), 8.11 (bs, 2H), 7.31 (d, *J* = 7.7 Hz, 1H), 7.17 (d, *J* = 7.9 Hz, 1H), 5.59 (s, 2H), 2.29 (s, 3H); ^13^C NMR (75 MHz, DMSO-*d*_6_) *δ* 154.1, 144.0, 143.5, 143.2, 139.4, 131.9, 129.7, 118.8, 104.3, 85.9, 82.9, 37.6, 21.5.

***6-Chloro-9-(4-methylbenzyl)-9H-purin-2-amine (14)***: A suspension of compound **2** (150 mg, 0.89 mmol), 4-methylbenzyl chloride (0.09 mL, 0.89 mmol), and K_2_CO_3_ (367 mg, 2.65 mmol) in DMF (1 mL) was stirred at rt for 9 h. The reaction solvent was evaporated under reduced pressure, and the residue was purified by column chromatography with dichloromethane/methanol (50/1, *v*/*v*) to afford the title compound (175 mg, 73% yield): ^1^H NMR (300 MHz, CDCl_3_) *δ* 7.75 (s, 1H), 7.30 (s, 1H), 7.20 (s, 4H), 5.24 (s, 2H), 5.18 (bs, 2H), 2.38 (s, 3H); ^13^C NMR (75 MHz, CDCl_3_) *δ* 162.3, 159.2, 153.9, 151.4, 142.2, 138.5, 132.1, 129.8, 127.8, 125.3, 47.0, 21.2; HRMS (FAB+) *m/z* calcd for C_13_H_13_ClN_5_ [M+H]^+^: 274.0859, found: 274.0857.

### 3.2. Biological Evaluations

#### 3.2.1. Fluorescence Polarization (FP) Assay Using Green BODIPY-GM and FITC-GM

FP measurements were performed on a FlexStation 3 microplate reader (Molecular Devices, Sunnyvale, CA, USA) using black 96-well microplates (Corning #3650) with a read time of 0.1s per well, following the procedure described earlier [42]. For the green BODIPY-GM, FP measurements were made with excitation at 485 nm (25 nm bandwidth) and emission at 530 nm (25 nm bandwidth) using a 505 nm beam splitter. The equilibrium binding experiments of fluorescent FITC-labeled geldanamycin (FITC-GM) to Hsp90α were conducted under the following conditions: in wells containing 5 nM FITC-GM, we added 30 nM human recombinant N-terminal Hsp90α protein (GenBank Accession No. NM_005348) in assay buffer (20 mM 4-(2-hydroxyethyl)-1-piperazineethanesulfonic acid (HEPES) (pH 7.5), 50 mM NaCl, 10 mM MgCl_2_, 0.02% Brij 35, 2 mM dithiothreitol (DTT), 0.02 mg/mL bovine serum albumin (BSA), 1% DMSO), and 5 nM FITC-labeled geldanamycin was used as a probe. The compounds in 100% DMSO were delivered into the enzyme mixture by acoustic technology (Echo550; nanoliter range) and were allowed to incubate for 30 min. FITC-GM mixture was delivered to initiate the reaction. The mixture was allowed to incubate for 3 h at room temperature with gentle mixing. Then, the FP was measured and mP values were calculated [41].

#### 3.2.2. Cell-Based Anticancer Evaluation by MTT Assay

Cytotoxic activity of certain Hsp90 inhibitors against human cancer cell lines (MCF-7, SK-BR-3, and HCT116) was investigated using the MTT assay following the reported procedure [35]. Cells were seeded into 96-well plates at a density of 3000 cells per well (MCF-7, HCT116, SK-BR-3 cells per each well). The compounds were added into cells and incubated for 72 h. Then, absorbance at 450 nm was measured using a Microplate Absorbance Reader (Bio-Rad, Hercules, CA, USA).

### 3.3. Biochemical, Cellular, and Structural Analysis 

#### 3.3.1. Expression and Protein Purification of *m*Hsp90ND 

Hsp90α (residues 9–225 Swiss Prot entry: P07901) gene was amplified by polymerase chain reaction (PCR) from *mouse* genomic DNA using *Pfu*Turbo polymerase (Stratagene, Sandiego, CA, USA). The PCR product was cloned into plasmid pET28a, which encodes a purification tag consisting of the amino acids His_6_ at the *N*-terminal of the protein. *m*Hsp90ND was expressed in *Escherichia coli* (*E. coli)* BL21 (DE3) codon plus RIL (Stratagene) cell. The cells were lysed by sonication in 50 mM Tris-HCl (pH 7.4), 100 mM NaCl, 2 mM 2-mercaptoethanol, and 0.2 mM phenylmethylsulfonyl fluoride (PMSF). After centrifugation, *m*Hsp90ND in the supernatant was purified by Ni^2+^-chelated Hi-trap chelating column, and HiLoad 26/60 200pg column (GE healthcare, Chicago, IL, USA). After the final step, *m*Hsp90ND was stored in 20 mM Tris-HCl (pH 7.4), 100 mM NaCl, and 1 mM dithiothreitol (DTT). 

#### 3.3.2. Binding Affinity Using ITC

ITC experiments were performed using the ITC200 instrument (Malvern Panalytical Inc., Westborought, MA, USA) at 298 K, and the data were analyzed using the program ORIGIN 7.0. The concentration of *m*Hsp90ND protein in the cell was 10–20 μM, while the syringe contained 200 μM of compounds **4b**, **6b**, **6c**, **14,** BIIB021, and 17AAG. Stock solutions of those compounds were prepared in dimethyl sulfoxide (DMSO) at 100 mM concentration and stored at −20 °C. The ITC buffer contained 20 mM Tris (pH 8.0), 100 mM NaCl, and up to 2% DMSO. Titrations were carried out at 25 °C, using 20 injections of 2 μL each, injected at 150 s intervals. No evidence for the binding of DMSO in the nucleotide-binding site was observed. The experimental raw data were corrected for dilution by subtracting the values for buffer alone and then fit by a one-site binding model. Titration data were fit using a nonlinear least squares curve-fitting algorithm with three floating variables: stoichiometry, binding constant (*K*_D_), and change of enthalpy of interaction. 

#### 3.3.3. Colorimetric Determination of ATPase Activity of Hsp90

In brief, ATPase activity assay was performed by mixing Hsp90α solution (5 μL, 6 μM in assay buffer), 20 μL assay buffer, ATP solutions (10 μL, 4 mM in assay buffer), and 5 μL of tested compounds (**14** and BIIB021) to yield final concentrations of 50 μM for 30 min at the room temperature using the QuantiChrom ATPase/GTPase Assay Kit (BioAssay Systems, Hayward, CA, USA). The reaction was terminated by adding 200 μL reagent buffer. Followed by incubation with reagent buffer for 30 min at the room temperature, the absorbance was measured at 620 nm using a Biotek-Synergy Neo-Plate Reader (BioTek Instruments, Winooski, VT, USA).

#### 3.3.4. Western Blot Assay 

MCF7 cells were seeded in 60 mm culture dishes, incubated overnight, and treated with the indicated concentrations of compound **14** or geldanamycin for 24 h. Cells were then harvested and lysed on ice by radioimmunoprecipitation assay (RIPA) buffer (23 mM Tris-HCl (pH 7.6), 130 mM NaCl, 1% nonidet P-40 (NP-40), 1% sodium deoxycholate, 0.1% SDS) supplemented with protease and phosphatase inhibitor cocktails, and 30 g of lysate perlane was separated by SDS-PAGE, followed by transferring to a polyvinylidene fluoride (PVDF) membrane (Bio-Rad, Hercules, CA, USA). The membrane was blocked with 5% skim milk in TBS-T, and then incubated with the corresponding primary antibody (EGFR, Akt, Hsp90, Hsp70, or GAPDH). After binding of an appropriate secondary antibody (Santa Cruz, CA, USA) coupled to horseradish peroxidase, proteins were visualized by enhanced chemiluminescence (ECL) according to the instructions of the manufacturer (GE healthcare, Chicago, IL, USA). 

#### 3.3.5. Crystallization and Data Collection 

The purified protein of *m*Hsp90ND was concentrated to 25 mg/mL in preparation for crystallization. Initial screening for the crystallization was carried out by using 96-well Intelli plates (Hampton Research) and Hydra II Plus One (MATRIX Technology Ltd, Toronto, Canada) robotics system at 295 K and optimized by the hanging-drop vapor diffusion in 24-well plates with a 2:1 mixture of protein solution and reservoir solution. Diffraction quality crystals of *m*Hsp90ND in complex with **4b**, **6b**, **6c**, **8**, and **14** were obtained using the hanging-drop vapor diffusion method by mixing an equal volume of protein solution with reservoir solution. For the inhibitor ternary complex, the inhibitor was added at a 1:5 molar ratio and incubated for 1 h at 277 K prior to crystallization. Diffraction quality crystals of the inhibitor complex were obtained by mixing 25 mg/mL protein solution and a reservoir solution with 1.0–2.0 M ammonium sulfate and 0.1 M Tris-HCl (pH 8.5) at 22 °C, with the crystals appearing in 1 day. All diffraction datasets were collected at a temperature of 100 K on the DSC Quantum 315 CCD area detector at the 5C SB-II beamline of the Pohang Light Source in Pohang, Korea. All datasets were processed using HKL2000. Statistics on the data are summarized in Table 4. 

#### 3.3.6. Structure Determination and Refinement

An initial model was obtained using the molecular replacement program MOLEP [46] with *h*Hsp90α (PDB code 1UY6) [47] as a model. Refinements were carried out using the programs CNS [48], CCP4 [49], and PHENIX [50], while model buildings were done using the program COOT [51]. Statistics on the final model are given in Table 4. The final structures were evaluated with the program Molprobity [52], and the figures were generated using Pymol (http://pymol.sourceforge.net/) and ESPript (http://espript.ibcp.fr/ESPript/ESPript/). The atomic coordinates and structure factors are deposited in the Protein Data Bank (www.pdb.org). The PDB ID codes are 7D24, 7D22, 7D1V, 7D26, and 7D25 for the **4b**, **6b**, **6c**, **8**, and **14** complex structures, respectively.

## 4. Conclusions

In this investigation, we report the synthesis and biological evaluation of a focused library of *N*^7^/*N*^9^-substituted purines as potential Hsp90 inhibitors. Our main objective was to explore the influence of replacing the 4-methoxy-3,5-dimethylpyridine, the hydrophobic binding moiety of BIIB021, with various simplified five-membered *N*/*O*-containing rings on π–π stacking interactions within pocket B of Hsp90 and the configuration of outer α-helix 4. Considering both biochemical and cell-based findings, the isoxazole derivatives **6b** and **6c** and the *p*-tolyl derivative **14** displayed promising Hsp90α inhibitory activity, with IC_50_ values of 0.203–1.76 µM. Moreover, the *p*-tolyl containing purine **14** elicited favorable anticancer potency over the Hsp90-relevant cancer cells MCF-7, SK-BR-3, and HCT116. The crystal structures and ITC of *m*Hsp90 *N*-terminal domain complexed with compounds **4b**, **6b**, **6c**, **8**, and **14** were determined, and their binding to the *N*-terminal domain of Hsp90 was pointed out. Furthermore, the structural findings revealed significance of conserving the aromaticity at *N*^9^-substituent of purine to make a proper angle between the purine ring and the aromatic ring (107°–110°) and achieve good binding affinity and hence considerable Hsp90α inhibitory activity. It could be concluded that the conformations of the protein atoms in the hydrophobic B pocket of Hsp90 remain constant in its ligand complex structure by tight binding and conserving outer helix for their biological activity. Considering these biological and crystallographic findings, we plan to extend our efforts in a further structural optimization study of BIIB021 based on binding mode and conformation of ligands at hydrophobic pocket B.

## Figures and Tables

**Table 1:**
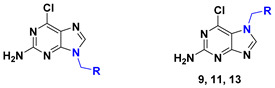
The competitive binding affinity of the target compounds against the fluorescein isothiocyanate labeled geldanamycin (FITC-GM) FITC-GM binding with Hsp90α by using fluorescence polarization (FP) assay.

Compound No.	R	Hsp90α	
		% Inhibition @10 μM ^a^	IC_50_, μM ^b^
**4a**	3-CH_3_CH_2_OCO-isoxazoline-	−2.4	- ^c^
**4b**	3-(CH_3_)_3_C-isoxazoline-	35.4	- ^c^
**6a**	3-CH_3_CH_2_OCO-isoxazole-	−4.7	- ^c^
**6b**	3-(CH_3_)_3_C-isoxazole-	95.8	1.76
**6c**	3-(CH_3_)_2_CH-isoxazole-	77.3	0.203
**7**	5-CH_3_CH_2_OCO-isoxazole-	14.8	- ^c^
**8**	2-C_6_H_5_-oxazole-	48.7	- ^c^
**9**	2-C_6_H_5_-oxazole-	NI ^d^	- ^c^
**10**	4-CH_3_-C_6_H_4_-C≡C-	22.2	- ^c^
**11**	4-CH_3_-C_6_H_4_-C≡C-	NI ^d^	- ^c^
**12**	5-(4-tolyl)-1*H*-1,2,3-triazole-	NI ^d^	- ^c^
**13**	5-(4-tolyl)-1*H*-1,2,3-triazole-	NI ^d^	- ^c^
**14**	4-CH_3_-C_6_H_4_-	95.4	1.00
BIIB021		96.1	0.029
17-AAG		100	0.056

^a^ The compounds were tested in a single dose (10 μM) duplicate mode against Hsp90α with FITC-GM, and the remaining percent of probe binding is shown. ^b^ The compounds were tested in a 10-dose IC_50_ with threefold serial dilution starting at 100 μM; IC_50_ values are the means of two independent experiments, and standard deviation (SD) is less than 20%. ^c^ Not determined. ^d^ NI: no inhibition was observed at 10 μM, and the tested compound exhibited a fluorescence background that might have interfered with the assay.

**Table 2:** In vitro antiproliferative activities of compounds **4b, 6b, 6c, 8,** and **14** against human cancer cells (MCF-7, HCT116, and SK-BR-3) ^a,b,c,d^.

Compound No.	MCF-7		SK-BR-3		HCT116	
	% Inh. at 10 μM	GI_50_ (μM)	% Inh. at 10 μM	GI_50_ (μM)	% Inh. at 10 μM	GI_50_ (μM)
**4b**	4.94	ND	13.47	ND	18.27	ND
**6b**	15.66	ND	**45.76**	26.03 ± 1.36	26.03	ND
**6c**	11.92	ND	**33.17**	ND	−0.60	ND
**8**	**33.27**	ND	**37.46**	22.54 ± 0.70	19.19	23.45 ± 2.94
**14**	**55.15**	16.22 ± 0.71	**60.93**	15.24 ± 2.08	**46.48**	ND
BIIB021	84.81	0.392 ± 0.03	71.94	0.347 ± 0.023	86.20	0.227 ± 0.015
17-AAG	79.74	0.498 ± 0.06	74.44	0.140 ± 0.012	88.89	0.205 ± 0.009

^a^ % inhibitions were obtained after incubation for 72 h using 3-(4,5-dimethylthiazol-2-yl)-2,5-diphenyl tetrazolium bromide (MTT) assay, and the values are the average of at least two independent measurements and SD are less than 20%. ^b^ Bold figures refer to growth inhibition >30%. ^c^ GI_50_ values are the means of two experiments ± standard error of the mean (SEM). ^d^ ND: not determined.

**Table 3:** ITC parameters for compounds **4b**, **6b**, **6c**, and **14** in *m*Hsp90ND at 25 °C.

Protein (in the Cell)	Ligand/Protein (in the Syringe)	N	*K*_D_ (µM)	∆H (Kcal/mol)	∆S (cal/mol/deg)
*m*Hsp90ND	**4b**	1.17	15.6	−19.8 ± 3.29	−44.5
	**6b**	0.97	0.44	−14.5 ± 0.4	−19.4
	**6c**	1.00	0.68	−15.8 ± 0.5	−24.8
	**14**	0.89	0.41	−17.8 ± 0.2	−30.5
	BIIB021	0.87	0.0083	−22.7 ± 0.2	−39.1
	17-AAG	0.98	2.9	−8.6 ± 0.4	−3.66

**Table 4:** Statistics on data collection and refinement of crystal structures of *m*HSP90ND complexed with compounds **4b, 6b, 6c, 8,** and **14**.

	4b	6b	6c	8	14
**Data Collection Statistics**					
X-ray source	PAL5-C				
Wavelength (Å)			1.0000		
Resolution range (Å)	50.0–1.55	50.0–1.60	50.0–1.33	50–1.75	50–1.65
	(1.61–1.55) ^a^	(1.66–1.60)	(1.38–1.33)	(1.81–1.75)	(1.71–1.65)
Space group	I222				
Unit cell parameters					
a (Å)	67.35	68.14	67.10	67.61	67.60
b (Å)	89.89	89.90	90.08	89.38	98.82
c (Å)	98.59	98.78	98.71	98.21	98.23
α β γ (°)	90	90	90	90	90
Total/unique reflections	854872/43890	786882/40417	1934188/68575	992546/30364	917934/36280
Completeness (%)	95.7 (82.8) ^a^	97.3 (92.5)	99.3 (98.0)	98.6 (96.2)	98.5 (96.5)
*I*/σ (*I*)	37.0 (4.3) ^a^	23.5 (1.9)	55.1 (4.3)	20.3 (2.9)	31.1 (3.3)
Redundancy	6.4 (4.2) ^a^	5.4 (3.0)	10.8 (7.0)	5.5 (3.4)	5.8 (3.9)
Rmerge ^b^ (%)	4.0 (20.6) ^a^	4.8 (34.1)	5.6 (32.8)	9.3 (27.7)	5.3 (25.2)
**Refinement Statistics**					
Resolution range (Å)	50.0–1.55	50.0–1.60	50.0–1.33	50–1.75	50–1.65
	(1.61–1.55) ^a^	(1.66–1.60)	(1.38–1.33)	(1.81–1.75)	(1.71–1.65)
*R*-value/Rfree ^c^ (%)	20.3/22.3	19.4/22.2	17.8/19.3	19.3/22.7	18.6/21.4
No. of protein atoms	1641	1633	1641	1641	1641
No. of water molecules	330	327	457	216	311
No. of ligand molecules	1	1	2	1	2
Average *B*-factor (Å^2^)	22.50	25.84	18.20	29.69	24.06
bond lengths (Å)/bond angles (°)	0.007/0.993	0.006/0.831	0.006/0.922	0.007/0.916	0.007/1.079
Ramachandran analysis (%)					
Favored	98.07	97.54	98.55	96.62	97.10
Allowed	1.93	2.46	1.45	3.38	2.90
Outliers	0	0	0	0	0
PDB ID codes	7D24	7D22	7D1V	7D26	7D25

^a^ Values in parentheses refer to the highest resolution shell. ^b^ R_merge_ = ∑_h_∑_i_|*I*(*h*,*i*)|<*I*(*h*)>|/∑_h_∑_i_ *I*(*h*,*i*), where I(*h,i*) is the intensity of the ith measurement of reflection h, and <*I*(*h*)> is the mean value of *I*(*h*,*i*) for all i measurements. ^c^ *R*_free_ was calculated from the randomly selected 5% set of reflections not included in the calculation of the *R*-value.

## Abbreviations

17-AAG	Allyl-aminogeldanamycin
ATP	Adenosine triphosphate
17-DMAG	17-[[(2-Dimethylamino)ethyl]amino]geldanamycin
DMF	Dimethylformamide
DMSO	Dimethylsulfoxide
DTT	Dithiothreitol
FP	Fluorescence polarization
GM	Geldanamycin
HIF-1α	Hypoxia-inducible factor 1α
HNSCC	Head and neck squamous cell carcinoma
HRMS	High-resolution mass spectra
Hsp90	Heat shock protein 90
ITC	Isothermal titration calorimetry
*m*Hsp90ND	*N*-terminal domain of mouse Hsp90α
MTT	3-(4,5-Dimethylthiazol-2-yl)-2,5-diphenyl tetrazolium bromide
NMR	Nuclear magnetic resonance
PCR	Polymerase chain reaction
PDB	Protein Data Bank
PMSF	Phenylmethylsulfonyl fluoride
SAR	Structure–activity relationship
TCEP	Tris(2-chloroethyl) phosphate
TMS	Tetramethylsilane
